# Smoking Cessation and Symptom Burden in Patients After Oncologic Surgery

**DOI:** 10.1001/jamanetworkopen.2025.22769

**Published:** 2025-07-23

**Authors:** Katherine K. S. Rieth, AnaPaula Cupertino, Michael Shen, Hongying Sun, Luke Peppone

**Affiliations:** 1 University of Rochester Medical Center, Rochester, New York

## Abstract

This cohort study assesses motivation to quit smoking, smoking cessation, and symptom burden during postoperative adjuvant treatment among patients with cancer.

## Introduction

Surgery is a mainstay of treatment for patients with cancer. Despite increased surgical risks, treatment toxic effects, and reduced treatment efficacy,^1,2^ up to 60% of patients who smoke at diagnosis continue to smoke.^3^ While guidelines advocate for integrating tobacco treatment into oncology care, referrals to cessation treatment are inconsistent and relapse rates high.^4^ This study assessed motivation to quit smoking, smoking cessation, and symptom burden during postoperative adjuvant treatment to inform cessation strategies throughout surgical cancer care and the potential for intervention in the perioperative setting.

## Methods

This cohort study used data from a nationwide prospective cohort study of 1003 patients with cancer enrolled through the University of Rochester Cancer Center National Cancer Institute Community Oncology Research Program across 20 community oncology locations.^5^ The study was approved by all site institutional review boards, and written informed consent was obtained from each participant before data collection. This study followed the STROBE reporting guideline.

Surveys were administered at 3 time points: before initiation of chemotherapy or radiation treatment, within 2 weeks after the completion of adjuvant treatment, and 6 months after the completion of treatment. Variables included smoking status and treatment-related side effects (via a 12-item inventory). Motivation to quit smoking was defined as planning to (1) decrease smoking, (2) quit after treatment, or (3) quit immediately. Race and ethnicity were self-reported (Black, White, or other [American Indian or Alaska Native, Asian, Hispanic or Latino, and Native Hawaiian or Other Pacific Islander]) and assessed to examine disparities in smoking status and cessation outcomes.

χ^2^ Tests, *t* tests, and analysis of variance were used to assess differences in sociodemographic characteristics, smoking status, motivation to quit, and symptom burden across time points. A significance level of *P* < .05 was used for all statistical comparisons. Data analyses were performed between September 1 to 30, 2024, using SPSS, version 30.0 (IBM Corp).

## Results

Of 1003 patients enrolled, 718 (71.6%; 526 female [73.6%] vs 158 male [22.0%]) underwent surgery (Table). Of these patients, 90 (12.5%) reported current smoking before treatment. Smoking before treatment was associated with younger age (<45 to 54 years, 46 patients [51.1%]; ≥55 years, 44 patients [48.9%]; *P* = .006) and higher proportions of Black participants (11 [12.2]) vs those of other race and ethnicity (3 [3.3%]) (*P* < .001). Among smokers, 62 (68.7%) had tobacco-related cancers. Symptom burden and symptom severity scores were significantly higher among patients who smoked vs those who did not smoke before treatment (23.7 vs 17.0 and 2.0 vs 1.4, respectively) (both *P* < .001) and at 6 months follow-up after treatment (33.5 vs 21.3 and 2.8 vs 1.8, respectively) (both *P* < .001) (Figure). Motivation to quit was sustained among patients currently smoking between the pre- and posttreatment periods. Although 58 smokers (64.4%) reported motivation to quit after surgery, only 14 (15.4%) quit before adjuvant therapy, and 29 (50.0%) relapsed by 6 months.

**Table.** Characteristics of Patients Who Did and Did Not Smoke Before Cancer Treatment

Characteristic	Patients, No. (%)		*P* value
	Smoked before treatment	Did not smoke before treatment	
Underwent surgical treatment	90 (15.1)	594 (86.8)	NA
Sex			
Female	67 (74.4)	459 (77.3)	.32
Male	23 (25.6)	135 (22.7)	
Age group, y			
<45	22 (24.4)	77 (13.0)	.006
45-54	24 (26.7)	118 (19.9)	
55-64	23 (25.6)	177 (29.8)	
65-74	12 (13.3)	151 (25.4)	
≥75	9 (10)	71 (11.9)	
Race and ethnicity			
Black	11 (12.2)	21 (3.5)	<.001
White	76 (84.4)	563 (94.8)	
Other^a^	3 (3.3)	10 (1.7)	
Cancer site			
Breast	46 (51.1)	370 (62.3)	.07
Genitourinary	13 (14.4)	70 (11.8)	
Lung	10 (11.1)	42 (7.1)	
Gastrointestinal	8 (8.9)	61 (10.3)	
Head and neck	0	7 (1.2)	
Gynecologic	11 (12.2)	30 (5.1)	
Hematologic	2 (2.2)	14 (2.4)	

Abbreviation: NA, not applicable.

^a^ Other races and ethnicities included American Indian or Alaska Native, Asian, Hispanic or Latino, and Native Hawaiian or Other Pacific Islander.

**Figure.**
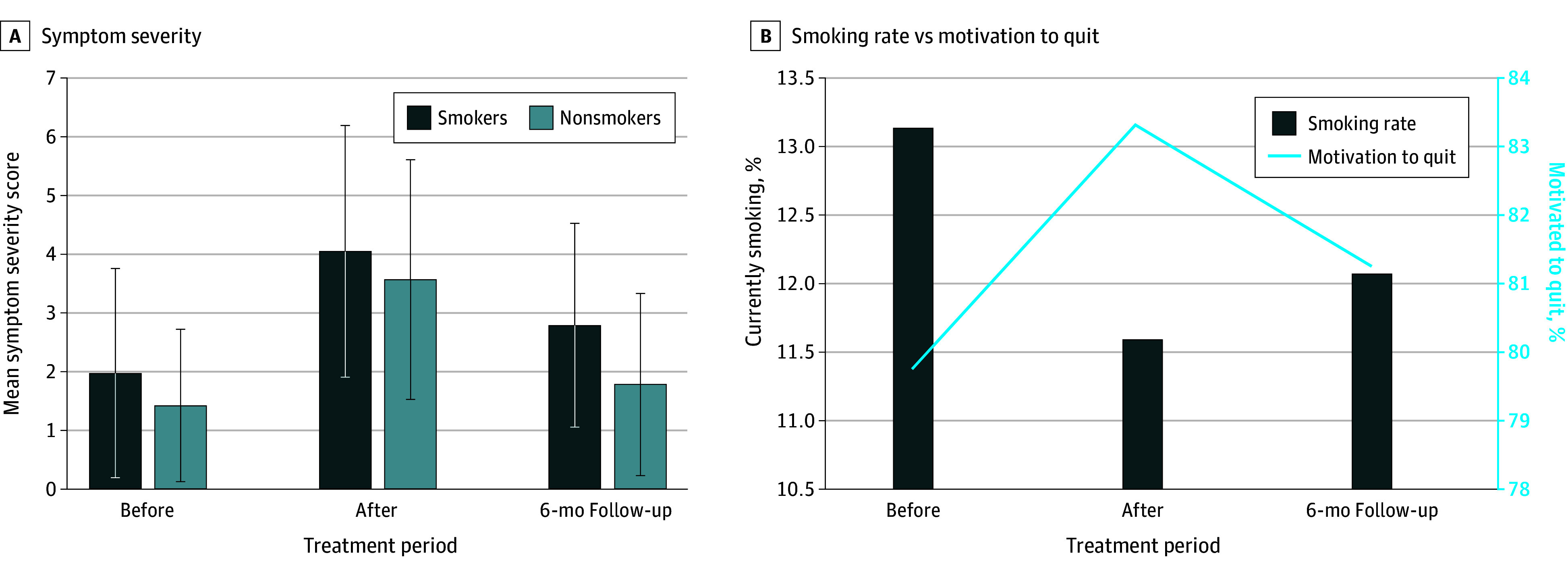
Symptom Severity, Smoking Rate, and Motivation to Quit After Oncologic Surgery Through Adjuvant Treatment Error bars indicate the SD.

## Discussion

The findings of this cohort study suggest that oncologic surgery may represent a unique teachable moment for smoking cessation while patients with cancer are highly motivated to quit. Clinical guidelines, individual motivation, and efficacy of cessation treatments are aligned, but quitting smoking during cancer treatment is complex. Without tailored approaches integrated into the continuum of cancer care, relapse may continue to be high, as identified in our study, which is consistent with reports of 70% of hospitalized smokers experiencing relapse by 6 to 12 months.^6^ As expected, patients who smoke experienced greater symptom burden during cancer treatment. Continued smoking after treatment led to significantly higher symptom severity scores at the 6-month follow-up, suggesting that ongoing smoking negatively affects quality of life.

Limitations of this study included reliance on self-reported smoking status and symptom measures and the inability to assess whether cessation interventions were received. Additional research is needed to evaluate integrated, longitudinal cessation programs in surgical oncology care to improve surgical and cancer-related outcomes.

## Notes

Supplement.
Data Sharing Statement
