# Supplementary material for: Smoking Cessation and Symptom Burden in Patients After Oncologic Surgery
Source: JAMA Netw Open. 2025 Jul 23;8(7):e2522769. doi: 10.1001/jamanetworkopen.2025.22769 (PMC12287831; doi:10.1001/jamanetworkopen.2025.22769)
Supplement: Supplement. — Data Sharing Statement [file jamanetwopen-e2522769-s001.pdf]

Data Sharing Statement

Rieth. Smoking Cessation and Symptom Burden in Patients After Oncologic Surgery. *JAMA Netw Open*. Published July 23, 2025. doi:10.1001/jamanetworkopen.2025.22769

Data
Data available: Yes
Data types: Deidentified participant data
How to access data: https://gitlab-public.circ.rochester.edu/4901/smoking_surgery.git
When available: With publication

Supporting Documents
Document types: None

Additional Information
Who can access the data: Anyone requesting the data
Types of analyses: For specified purpose
Mechanisms of data availability: With signed data access agreement
